# O-55 - NHS SCOTLAND’S JOURNEY TO REDEFINING RESPIRATORY TRANSMISSION: THE COVID-19 PANDEMIC’S LEGACY

**DOI:** 10.1093/pubmed/fdag046.049

**Published:** 2026-07-09

**Authors:** Ms Lorna Gordon, Ms Sofie French, Ms Emma Hooker, Ms Anna Munro, Ms Leighanne Bruce, Ms Yasmine Lutton, Dr Lauren Blane, Ms Susie Dodd

**Affiliations:** ARHAI Scotland, National Services Scotland; ARHAI Scotland, National Services Scotland; ARHAI Scotland, National Services Scotland; ARHAI Scotland, National Services Scotland; ARHAI Scotland, National Services Scotland; ARHAI Scotland, National Services Scotland; ARHAI Scotland, National Services Scotland; ARHAI Scotland, National Services Scotland

## Abstract

**Background:**

The COVID-19 pandemic prompted a re-evaluation of the historic concepts underpinning respiratory infection prevention and control (IPC) guidance, inclusive of the ‘droplet/airborne’ dichotomy and aerosol generating-procedures. In 2022, ARHAI Scotland initiated a systematic literature review on Transmission Based Precautions (TBPs), published in the Scottish National Infection Prevention and Control Manual (NIPCM) in August 2024. A key research focus was exploration of how infectious agents are emitted from the respiratory tract. The review concluded that traditional transmission definitions do not reflect the continuum of infectious particle sizes that can be found close to, and at distance from, a source, and that many procedures classified as AGPs may not pose a greater risk than exposure to coughing.

**Objectives:**

Develop IPC guidance aligned with findings of the TBP definitions systematic literature review, informed by multidisciplinary expert opinion and reflecting lessons learned from the COVID-19 pandemic.

**Methods:**

An ARHAI Scotland working group was convened in April 2024. Members of the multidisciplinary group included representatives from IPC teams across 14 regional NHS Boards, Dentistry, Primary Care, the Scottish Ambulance Service, Health and Safety, Occupational Health, Public Health and NHS Education for Scotland. Evidence was reviewed across more than 20 meetings, with additional input from experts in aerosol science and respiratory medicine.

**Results:**

Guidance changes include discontinuation of the terms ‘droplet’, ‘airborne’ and ‘AGP’, classification of respiratory pathogens by severity of infection and introduction of a risk-based TBP assessment which considers pathogen, proximity, presence of symptoms, procedures, occupational health risk and personal choice.

**Conclusions:**

ARHAI Scotland is the first IPC body globally to apply new descriptors to operational IPC guidance. A phased implementation is planned with a soft launch of guidance changes in the spring, and full national implementation by the end of summer.

